# Recognition memory, primacy vs. recency effects, and time perception in the online version of the fear of scream paradigm

**DOI:** 10.1038/s41598-022-18124-9

**Published:** 2022-08-22

**Authors:** Armin Zlomuzica, Fine Kullmann, Julia Hesse, Laurin Plank, Ekrem Dere

**Affiliations:** 1 Department of Behavioral and Clinical Neuroscience, Ruhr-University Bochum (RUB), Massenbergstraße 9-13, 44787 Bochum, Germany; 2 UMR 8256: Adaptation Biologique Et Vieillissement, Sorbonne Université. Institut de Biologie Paris-Seine, (IBPS), UFR Des Sciences de La Vie, Campus Pierre et Marie Curie, Bâtiment B, 9 quai Saint Bernard, 75005 DépartementParis, France

**Keywords:** Neuroscience, Psychology, Psychiatric disorders

## Abstract

Anxiety disorders are characterized by cognitive dysfunctions which contribute to the patient’s profound disabilities. The threat of shock paradigm represents a validated psychopathological model of anxiety to measure the impact of anxiety on cognitive processes. We have developed an online version of the threat of scream paradigm (ToSP) to investigate the impact of experimental anxiety on recognition memory. Two animated passive walkthrough videos (either under threat of scream or safety conditions) were shown to healthy participants. Recognition memory, primacy vs. recency effects, and subjective estimations of the length of encoding sessions were assessed. Subjective anxiety, stress, and emotional arousal ratings indicated that experimental anxiety could successfully be induced (Safe-Threat) or reversed (Threat-Safe) between the two passive walkthrough sessions. Participants exposed to distress screams showed impaired retrieval of complex information that has been presented in an animated environment. In the threat condition, participants failed to recognize details related to the persons encountered, their spatial locations, as well as information about the temporal order and sequence of encounters. Participant groups, which received a threat announcement prior to the first walkthrough session (Threat-Threat vs. Safety-Safety and Threat-Safety vs. Safety-Threat) showed poorer recognition memory as compared to the groups that received a safety announcement (*P* = 0.0468 and *P* = 0.0426, respectively; Mann–Whitney U test, Cohen’s *d* = 0.5071; effect size *r* = 0.2458). In conclusion, experimental anxiety induced by the online version of the ToSP leads to compromised recognition memory for complex multi-dimensional information. Our results indicate that cognitive functions of vulnerable populations (with limited mobility) can be evaluated online by means of the ToSP.

## Introduction

Anxiety disorders are highly common and belong to the most debilitating mental disorders^1^. Characteristic symptoms of anxiety disorders comprise a sense of impending dread, danger or threat, hyperarousal and irritability, pervasive worrying, avoidance, as well as cognitive dysfunctions which contribute to the maintenance and chronification of these symptoms^2,3^.

The integration of new learning experiences within existing memory networks during cognitive behavioral therapy for anxiety disorders is essential for a successful therapy outcome^4^. This, however, might be hampered due to changes in cognitive processing in patients with anxiety disorders, i.e. biased attention^5^ and/or alterations in learning and memory functions^6–10^. Thus, a better understanding of cognitive alterations in the context of pathological anxiety may ultimately lead to the development of more mechanistic-based therapeutic approaches for anxiety disorders^5,11–17^.

It is well known that emotions can modulate learning and memory formation^7^. In a similar vein, anxiety can compromise complex information processing and storage in healthy and aged individuals, as well as in patients with anxiety disorders^6,8,10,18–22^. The threat of shock paradigm has been developed to model the hyperarousal and diffuse feelings of danger or threat which are characteristic for patients with pathological anxiety to investigate the impact of anxiety on basic cognitive and behavioral processes including perception, attention, concentration, face recognition, spatial and verbal working memory, decision making, impulsivity or defensive reflex activity^23–27^.

Recent evidence suggests that experimental anxiety induced by threat instructions reliably affects face recognition memory. Experimental anxiety induced by verbal threat instructions impairs face identity recognition in an unexpected item/source recognition task^28^. Similarly, threat of shock impairs the recognition of faces paired with a negated personality descriptor (signaled to be untrue) as compared with a verified personality descriptor (signaled to be true)^29^. These findings suggest that experimental anxiety impairs the encoding and processing of social stimuli.

Recently the threat of scream paradigm (ToSP) has been developed as an alternative to the threat of shock paradigm, to be used with more vulnerable study participants^30^. Using skin conductance measurements, it has been shown that the ToSP is effective in inducing experimental anxiety in a consistent and sustained manner^30^. However, data on the utility of the ToSP for the investigation of complex cognitive processes, including multidimensional recognition memory has not yet been provided.

We have developed an online version of the ToSP that can be performed by participants outside the laboratory and investigated, whether this online version is indeed suitable to induce experimental anxiety in healthy individuals. Furthermore, the effects of experimental anxiety induction on complex recognition memory, primacy vs. recency position effects, as well as on the estimation of the duration of information encoding sessions has been investigated.

## Methods

### Participants

Participants, aged between 18 and 50 years, were recruited via the university’s research study website and social media platforms. Prior to the beginning of the experiment the participants had to indicate whether they suffer from acute or chronic neurological and/or psychological illness. All participants reported normal or corrected-to-normal vision. Further exclusion criteria were acute prescription-only medication or substance abuse. A total of 141 participants consented to participate in the study. Ninety-four participants (64.83%) completed the online experiment. As scheduled prior to the recruitment of the participants, those participants who finished the experiment before 25 min or did not complete the study after 90 min had elapsed, were excluded from the study. Statistical analysis was then performed with a final dataset that comprised eighty-seven participants.

All experimental procedures have been approved by the ethics committee of the Ruhr University of Bochum. The study has been performed in accordance with the Declaration of Helsinki. The general information’s for the participants introduced the study as an *investigation that aims to determine the effect of emotions and stress on visual perception in an animated scenario*. This misleading information about the purpose of the study was given to avoid intentional encoding of the content of the passive walkthrough videos and to promote incidental encoding that might be modulated by emotional arousal. All participants of the online study provided informed consent and were compensated for their participation with Amazon gift cards (10€).

### Experimental design and procedures

**Figure 1:**
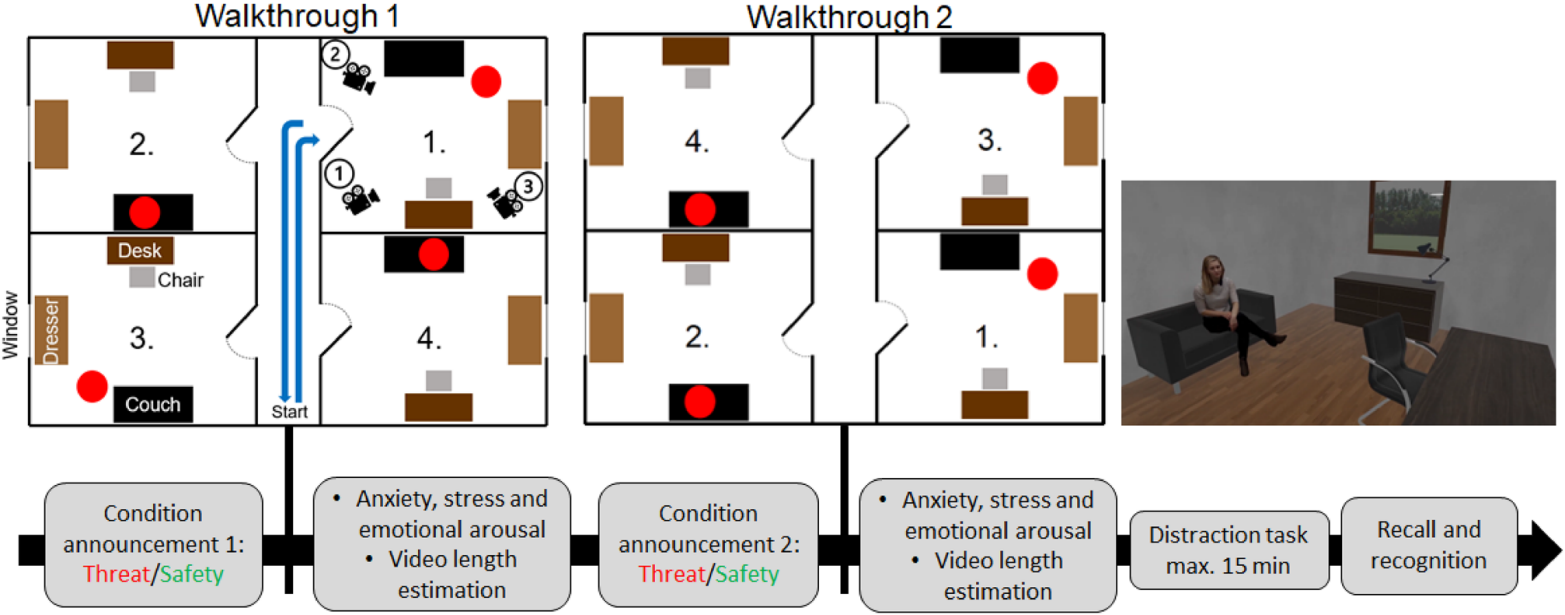
General experimental design and procedures. After the announcement of the presence or absence of distressing screams, the passive walkthrough 1 begun. The tracking shot started in the hallway and entered the rooms in ascending order (1–4). In each room the camera positioned itself at three locations in ascending order (marked by the circled numbers 1–3). Before the camera entered a new room, it returned to the starting point in the hallway. The red dot indicates the position of the person stimuli in the room. The person stimuli were either standing between the couch and the dresser or sitting on the couch. Apart from the person stimuli, all rooms were arranged identically. The rightmost picture depicts an exemplary room. After walkthrough 1 the participants were asked to rate their anxiety, stress and emotional arousal on a 9-point Likert scale and to estimate the length of the walkthrough. This process was repeated for walkthrough 2, which was similar to walkthrough 1, but differed in terms of the position of the person stimuli in the rooms and the route of the tracking shot. After the walkthroughs, the subjects engaged in a distraction task. After the distraction task, the subjects were asked to perform a memory recall and recognition test.

The experiment was performed online using a web based (Qualtrics) survey tool. Figure 1 shows a general overview of the experimental procedures and manipulations in the order in which the subjects went through the different parts of the experiment. The participants were recommended to use a desktop or laptop computer for the online experiment rather than a device with a small display. Before the start of the experiments the participants were asked to complete the depression, anxiety and stress scale version 21 (DASS-21)^31^.

### Anxiety, stress and emotional arousal ratings

At the beginning of the online experiment the participants were asked to rate their current level of anxiety, stress and emotional arousal using visual analogue scales with ranging from 1 = not at all to 9 = very much. These measurements served as manipulation checks that served to ensure the efficacy of the online version of the ToSP. Thereafter, the participants were asked to listen to four different audio recordings of distress screams and to adjust the loudness of their device speakers to an unpleasant but still tolerable intensity. Then the participants had to indicate which one of the four distress screams induced the highest level of discomfort/aversiveness. Thereafter, the selected distress scream recording was rated on a scale ranging from 1 = not at all unpleasant to 9 = very unpleasant.

### Instructions

In the following the participants were informed that they are about to see videos and that there is a probability that the distress scream, which has been rated as most unpleased, might be played at any moment without warning. The participants were instructed that an announcement will be made prior to the beginning of the videos. The announcement will be indicative of whether there is a risk to be exposed to the distress scream (threat) or not (safety).

### Experimental conditions

The participants were randomly assigned to one of four experimental conditions. The control group went through both passive walkthrough videos under the safety condition (Safety-Safety group: Incidental memory encoding without emotional arousal induction). The experimental group went through both passive walkthrough videos under the threat condition (Threat-Threat group: Incidental memory encoding with emotional arousal induction). In the two mixed condition groups (Safety-Threat and Threat-Safety groups), the sequence of conditions (safety first-threat second vs. threat first-safety second) was randomized across the participants assigned to the mixed conditions. The effectiveness of the online version of the ToSP to modulate subjective measures of emotional activation, primacy and/or recency effects on memory, time perception and estimation, as well as recognition memory was assessed through comparisons between the Safety-Safety and Threat-Threat groups. Comparisons between the Safety-Threat and Threat-Safety groups were performed in order to investigate the dynamics and time-course of the emotional activation. Specifically, the mixed groups were compared to detect possible carry-over effects, to have an estimation of the responsiveness/rapidity of the emotional system after a threat announcement, and to test whether the emotional activation induced by threat would be immediately reversible after a safety announcement. Demographic characteristics of the groups are listed in Table 1.

**Table 1:** Demographic characteristics of randomized assignment of participants to control and experimental groups. No significant group differences were observed with respect to the parameters listed below. P-Values given refer to Kruskal–Wallis statistics. Abbreviations: (S-S: Safety-Safety condition; T-T: Threat-Threat condition; S-T: Safety-Threat condition, T-S: Threat-Safety condition. Values listed refer to medians and interquartile ranges; N = Sample size; M = Male, F = Female, D = Divers; Education = Highest graduation or academic degree: Lowest = 1:No graduation, Highest: 8: Doctorate degree; DASS: Depression Anxiety Stress Scales; n.s. = not significant).

	S-S	T-T	S-T	T-S	Group comparisons
N =	22	22	22	21	
Gender	M:18, F:3, D:1	M:10, F:12, D:0	M:13, F:9, D:0	M:11, F:10, D:0	*P* = 0.1202, n.s
Age	24 [23, 25]	25 [24, 30]	28 [25.25,32.25]	25 [23, 29]	*P* = 0.0755, n.s
Education	5 [5, 6]	5 [5, 6]	5 [5, 6]	5 [5, 6]	*P* = 0.8855, n.s
DASS Depression	3 [0,7]	4 [1.25,3.5]	4 [2,5.75]	3 [2, 4]	*P* = 0.5662, n.s
DASS Anxiety	1 [0,3]	2.5 [0.25,5]	2 [1,4.75]	2 [1, 5]	*P* = 0.7421, n.s
DASS Stress	5,5 [1.25,9]	6 [3.25,9.75]	2.5 [2, 8]	7 [4, 10]	*P* = 0.5056, n.s

### Scream exposure announcements

The announcements were randomized across the participants of the mixed groups and were as follows: “You are now at risk to be exposed to distress screams”, or “You are safe from being exposed to distress screams”. Actually, the distress screams were never administered no matter what announcement was presented. The videos consisted of animated passive walkthrough videos which led through four rooms in which four different persons were present (for a detailed description of the videos see the section “Passive walkthrough videos”).

After each video the participants were again asked to rate their current level of anxiety, stress and emotional arousal using visual analogue scales in order to evaluate whether the threat of scream condition would lead to an increase in these measures.

### Time perception and estimation

Previous research has suggested a linear correlation between the subjective assessment of the length of a video presentation containing information to be encoded and the performance in a subsequent memory test for the information presented during the video. Therefore, the participants were requested to give an estimation of the duration of each walkthrough video. It has been suggested that the subjective estimation of the length of a learning trial can be considered as a predictor/signature of coding efficiency and thus memory performance^32^. The length estimation was made on a visual analogue scale ranging from 0 to up to 510 s. This measure was implemented to detect whether emotional arousal would have an effect on subjective time length assessment and whether the perceived length of a video might be correlated with recognition performance.

#### Sociodemographic questionnaire

After the two video presentations, the associated anxiety, stress and arousal self-reports, as well as the subjective time assessments, the participants were given a short sociodemographic questionnaire before they moved on to an item categorization task.

### Distraction task

The distractor item categorization task lasted between 10 and 15 min to prevent the rehearsal of information presented during the videos as well as possible recency effects on the recognition parameter. The task consisted of the presentation of 300 images. The participant was requested to indicate, (with a maximal response latency of 3 s before the next image was automatically presented), whether the stimuli presented were either alive or unanimated objects.

### Test for primacy or recency effects

After the completion of the distractor task the participants were asked whether they spontaneously (without the presentation of specific retrieval cues that were taken from the videos) remember an “encounter” from the two videos. This was done to check whether the distractor task was effective in blocking a pronounced recency effect with respect to the content of the videos. The participants were given 90 s to provide details of a specific encounter they could spontaneously remember, without being explicitly asked to indicate which specific person they have encountered. Details to be reported included the specification of the video (first or second), the exact position in the sequence of the encounters (first, second, third, fourth), the posture, hair color and gender of that person. Finally, participants had to indicate whether this encounter was made under the risk of being exposed to distress screams or under the safety condition. In the case that the participants stated that they do not spontaneously remember further encounters they could pass on to the recognition tests. The recency check was performed to know whether the participants would remember the last encounter in the second video more frequently as compared the other encounters from both the first and second video. This information was important to ensure that the recognition tests performed thereafter were not based solely on working or immediate memory.

### General procedure of the recognition assessments

In the following the recognition memory of the participants was recorded by presenting them with close-up images of persons who were seen during the videos or similar images showing unfamiliar persons. During a series of different recognition tests the participants had to discriminate persons from the videos from novel persons, indicate in which room and in which position within the sequence of encounters that specific person was encountered and whether the encounter was made under the treat or safety condition (For further details see the section termed “Detailed procedure of the recognition assessment” below). After the completion of the recognition tests the participants had to indicate which device was used to listen to the distress screams and for watching the videos. Furthermore, the participates had to indicate whether they have made an attempt to memorize information during the presentation of the videos, whether they have paid attention to the duration of the videos, whether they have expected a memory test at the end of the study and finally whether they have encountered any difficulties in understanding the questions, experienced distractions or technical problems throughout the study. At the end of the experiment, the subjects were informed of the true purpose of the study and received their desired participation compensation.

### Screams stimuli

Four different distress screams with male and female voices were used in the online study. The distress screams audio files with the numbers 275, 276, 277, and 292 were taken from the International Affective Digitized Sounds database^33^. The effectiveness of screams to induce emotional arousal and to affect stress-related electrodermal activity has allready been demonstrated by Beaurenaut and colleagues^30^.

### Passive walkthrough videos

The stimuli presented during the two videos have been generated based on characteristics of previously used encoding material that has been proven to be appropriate for memory tasks^8,21^. The animated environment in which these stimuli have been embedded was created using the software Blender 2.91.2 and designed with 3D person models from the BlenderKit database. The animated environment consisted of a central hallway from which four rooms were accessible (two rooms on each side of the hallway). One 3D model person was placed in each room either in a sitting position or standing upright. Each passive walkthrough video involved two persons in a sitting position and two more persons standing in an upright position. Six different person stimuli were used for the videos. Two person stimuli already presented during the first video reappeared during the second video. These “familiar” person stimuli were presented with a different posture and appeared at a different position in the temporal sequence of the four encounters/room entries. This reappearance of familiar person stimuli in a slightly different context was intended to induce memory interference and increase the difficulty of the recognition task. Each room of the animated environment was identical in its design and furnishing. It contained one desk, chair, couch and one dresser with a lamp on it. At the wall opposite to the entry a window was placed through which a generic green floor and green trees were visible. Person stimuli standing upright were always located between the couch and the dresser, while the other person stimuli were always seated on the couch. However, the location of the person stimuli in a specific room differed between walkthrough 1 and 2. For example, in the bottom right room in walkthrough 1 the person stimulus is sitting on a couch, while in the bottom right room in walkthrough 2 the person stimulus is standing between the dresser and the couch. The experimental conditions (Threat vs. Safety) did not affect the person stimulus locations in the rooms. During the passive walkthrough each room was visited by the participant for a total duration of 45 s during which the participant could explore the room from three different angles following a tracking shot through the room. Before walking to the next room, the camera always moved back to the original position (hallway) prior to the start of the next tracking shot. The sequence in which the rooms were entered was different for the two passive walkthroughs/videos. Both videos had a fixed duration of 4 min and 12 s. The sequence in which the two videos were presented was randomized across participants. An overview of the experimental design is illustrated in Fig. 1.

### Detailed procedure of the recognition assessment

Participants were shown images of persons within the animated environment and their memory for the information presented in the first and the second video was examined. The participants were asked to remember details of the person stimuli presented and the context of their appearance. The participants were asked to categorize the images into familiar (target stimuli) or unfamiliar (non-target stimuli) depending on, whether the content was shown in one of the previous videos or not. Participants were instructed to pay attention to the persons or characters depicted, as well as to their clothes and posture. The images that were shown in a random order and included all eight encounters from both videos (target stimuli) as well as eight distractor images (unfamiliar or non-target stimuli). The non-target stimuli were matched with the target images with respect to the number of persons sitting or standing and their gender. Three of the unfamiliar or non-target stimuli showed familiar persons (encountered during the videos), who, however, were dressed differently. The remaining five unfamiliar or non-target stimuli instead showed unknown persons that were not encountered in the videos. The participants were also asked to remember the circumstances of the target stimuli presented during the two passive walkthrough videos. The participants had to indicate whether each of the 8 target stimuli was presented after the “safety” or “threat” announcement. Thereafter, the participants had to specify the room where the person stimuli were presented. A layout of the hallway and the rooms labeled from one to four was shown together with every image depicting a person stimulus. Furthermore, participants had to specify whether a person stimulus was encountered as the first, second, third or last person stimulus within the temporal sequence of each video and had to indicate the sequence in which the four rooms were entered in the first and second video.

### Recognition data analysis

The recall and/or recognition of information that has been encoded during walkthrough 2 might be more difficult, because half of the person stimuli have been reused after their initial presentation during walkthrough 1. This was done in order to avoid ceiling effects and to increase the difficulty of the recognition task for healthy participants. However, this manipulation might have led to a higher susceptibility to interference during the recall and/or recognition of information encoded during walkthrough 2. Furthermore, given that screams were not administered during walkthrough 1, the emotional activation response to the threat announcement in the Threat-Threat group before walkthrough 2 might have been weaker as compared to the emotional activation induced by the first announcement. Therefore, data from walkthrough 1 and 2 have been analyzed separately. Total recognition scores for information retained from the 2 passive walkthrough videos included correct identification of target stimuli (score range 0–4) as well as correct responses to the spatial–temporal context of the 4 target stimuli with respect to the experimental condition threat vs. safety, the spatial location of the target stimuli, the particular position in the temporal sequence of the appearances (0–12).

### Statistical procedures

The data shown in figures and text are presented as median ± interquartile range. Between- and within group comparisons were made for total recognition scores of walkthrough 1 and 2 separately. Between group comparisons were made either with Kruskal–Wallis tests or Mann–Whitney U-tests. Effect-sizes of significant pairwise between-group comparisons were determined with Cohen’s *d* and *r* effect-sizes. Within-group comparisons were made with Wilcoxon matched-pairs signed rank tests. *P*-values reported are two-tailed and considered to be significant at the alpha-level of *P* < 0.05 for Kruskal–Wallis tests and Mann–Whitney U-tests. Wilcoxon pairwise tests were considered significant at the alpha-level of *P* < 0.025.

## Results

### Threat of scream manipulation check

The validity of the online version of fear of scream paradigm was analyzed using the sum score of subjective anxiety, stress and emotional arousal assessments that have been recorded prior to the presentation of the first passive walkthrough video (Baseline measurement), and after the presentation of the two passive walkthrough videos.

**Figure 2:**
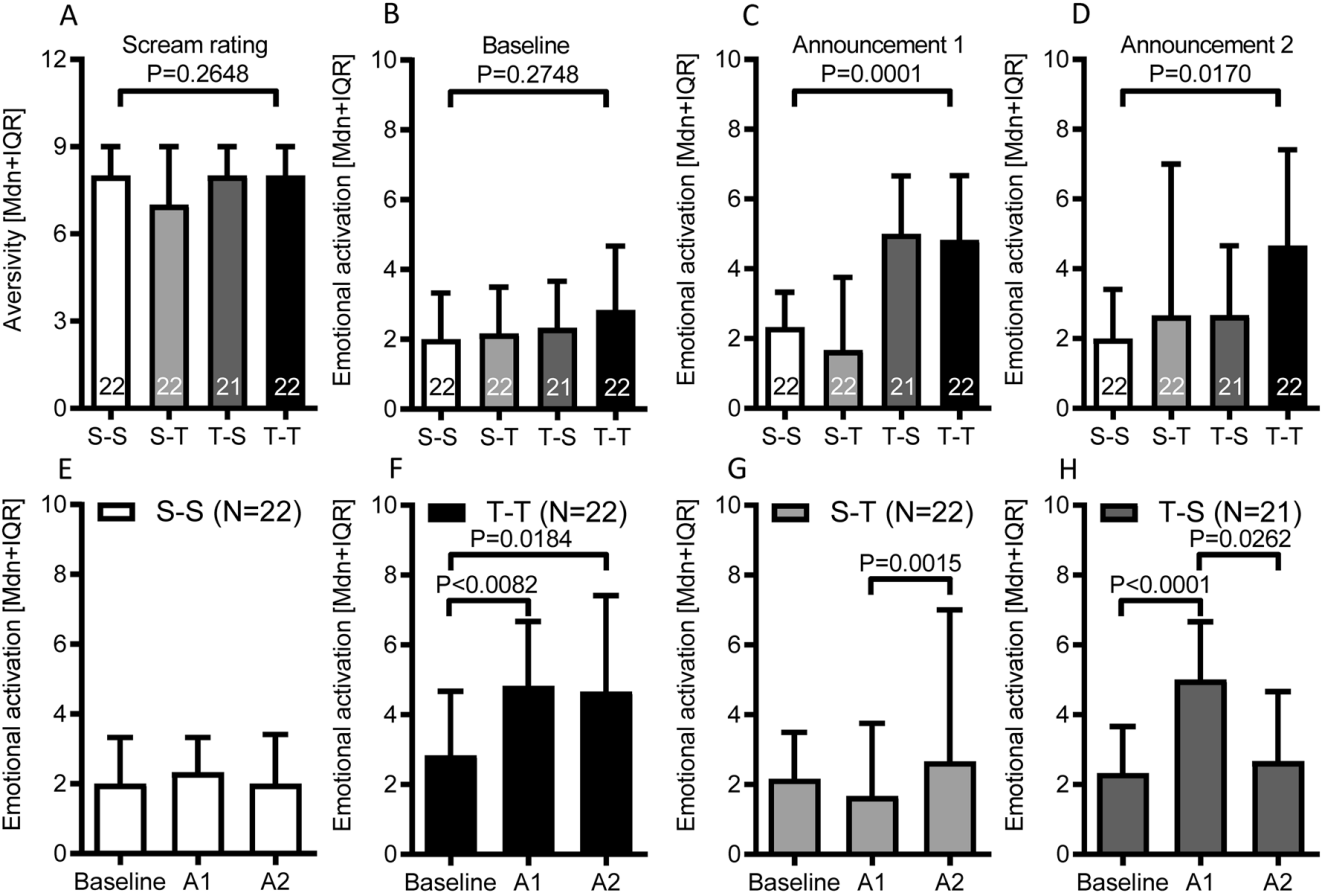
Validation of the online version of the threat of scream paradigm as a tool to induce experimental anxiety. (**A**) Distress scream rating: Subjective rating of the aversiveness of the selected distress scream on a visual analogue scale. (**B–D**) Emotional activation: Sum scores of subjective ratings (on visual analogue scales) of the current level of anxiety, stress and emotional arousal prior to the start of the experiment (Baseline), after the first or second announcement of threat or safety conditions. Each bar represents the median and interquartile range [Mdn + IQR] of corresponding visual analog scale ratings. Numbers within the bars indicate the sample size of the experimental condition. *P*-values given refer to Kruskal–Wallis tests for independent samples. Abbreviations: S-S: Safety-Safety condition; T-T: Threat-Threat condition; S-T: Safety-Threat condition; T-S: Threat-Safety condition. (**E–H**) Within-group dynamics of emotional activation during the baseline measurement and after threat or safety announcements for the four experimental conditions. Each bar represents the median and interquartile range [Mdn + IQR] of corresponding visual analog scale ratings. *P*-values given refer to Wilcoxon signed rank tests for dependent samples. Abbreviations: S-S: Safety-Safety condition; T-T: Threat-Threat condition; S-T: Safety-Threat condition; T-S: Threat-Safety condition.

The subjective aversiveness rating of the distress scream selected was not significantly different between the four groups (χ2[3] = 3.969, *P* = 0.2648; Kruskal–Wallis test; Fig. 2A). Furthermore, no significant between-groups differences were found for the baseline assessment of emotional activation (χ2[3] = 3.879, *P* = 0.2748; Kruskal–Wallis test; Fig. 2B). These results suggest that the subjective evaluation of the distress scream, as well as the subjective emotional state prior to threat or safety announcements, was comparable across groups and experimental conditions. As hypothesized, the reported emotional activation (that has been measured immediately after the end of the passive walkthrough videos) after the first and second threat vs. safety announcements was significantly different across experimental conditions (Announcement 1: χ2[3] = 21.06, *P* < 0.0001; Announcement 2: χ2[3] = 10.10, *P* = 0.0170; Kruskal–Wallis test; Fig. 2C,D).

### Emotion induction in the safety-safety and threat-threat groups

As expected, within-group comparisons between the 3 measurements (Baseline vs. Announcement 1: W = 27; *P* = 0.5382; Baseline vs. Announcement 2: W = 8; *P* = 0.8732; Announcement1 vs. Announcement 2: W = − 21; *P* = 0.6614; Wilcoxon test; Fig. 2E) indicated that the subjective emotional activation induced by the two Safety announcements was not significantly different from the baseline measurement in the Safety-Safety group.

The emotional activation of the Threat-Threat group was significantly higher after the first and second announcements relative to the baseline condition (Baseline vs. Announcement 1: W = 148; *P* = 0.0082; Baseline vs. Announcement 2: W = 143; *P* = 0.0184; Wilcoxon test; Fig. 2F). No significant difference was found for the comparison of the emotional activation induced by the first and second announcement. (Announcement1 vs. Announcement 2: W = 28; *P* = 0.6387; Wilcoxon test; Fig. 2F).

The subjective emotional activation during the baseline measurement was not significantly different between the Safety-Safety and Threat-Threat groups (U = 167; *P* = 0.0779; Mann–Whitney U test; Fig. 2E,F). As expected, the emotional activation induced by the first announcement was significantly higher in the Threat-Threat group as compared to the Safety-Safety group (U = 118; *P* = 0.0029; Mann–Whitney U test; Cohen’s *d* = 0.9866; effect size *r* = 0.4424; Fig. 2E,F). Similarly, after the second announcement, the subjective emotional activation was significantly higher in the Threat-Threat group as compared to the Safety-Saftey group (U = 112.5; *P* = 0.0018; Mann–Whitney U test; Cohen’s *d* = 1.0221; effect size *r* = 0.4551; Fig. 2E,F).

### Emotion induction in the mixed safety-threat and threat-safety groups

A within-group comparison of the Safety-Threat group indicated that the emotional activation measured during the baseline assessment was not significantly different from the one induced by the first announcement (no risk of distress scream exposure) (Baseline vs. Announcement 1: W = − 46; *P* = 0.3652; Wilcoxon test; Fig. 2G). The emotional activation after the second announcement (being at risk to be exposed to distress screams) was significantly higher as compared to the activation measured after the first announcement (Announcement 1 vs. Announcement 2: W = 136; *P* = 0.0015; Wilcoxon test; Fig. 2G). No significant difference was found for the comparison of the baseline measurement with the emotional activation induced by the second announcement (Baseline vs. Announcement 2: W = 77; *P* = 0.1856; Wilcoxon test; Fig. 2G). These results suggest that the emotional activation induced by a threat announcement leads to a weaker emotional response if preceded by a safety announcement.

The emotional activation of the Threat-Safety group was significantly higher after the first announcement (being at risk to be exposed to distress screams) relative to the baseline condition (Baseline vs. Announcement 1: W = 136; *P* = 0.0015; Baseline vs. Announcement 2: W = 219; *P* < 0.0001; Wilcoxon test; Fig. 2H). A trend for a significant difference was observed in comparison to the emotional activation measured after the second announcement (Announcement1 vs. Announcement 2: W = − 127; *P* = 0.0262; Wilcoxon test; Fig. 2H). No significant difference was found for the comparison of the emotional activation induced by the first and second announcement. (Baseline vs. Announcement 2: W = 70; *P* = 0.1649; Wilcoxon test; Fig. 2H). The emotional activation pattern of the Threat-Safety group, unlike the one exhibited by Safety-Threat group, suggests that the emotional activation induced by a threat announcement is reversible.

The subjective emotional activation during the baseline measurement was not significantly different between the Safety-Threat and Threat-Safety groups (U = 208.5; *P* = 0.5888; Mann–Whitney U test; Cohen’s *d* = 1.1576; effect size *r* = 0.5010; Fig. 2G,H). As expected, the emotional activation induced by the first announcement was significantly higher in the Threat-Safety group as compared to the Safety-Threat group (U = 92; *P* = 0.0005; Mann–Whitney U test; Fig. 2G,H). In contrast, after the second announcement, the subjective emotional activation was not significantly different between the Safety-Threat and Threat-Safety group (U = 225; *P* = 0.8896; Mann–Whitney U test; Fig. 2G,H).

In sum, the above results suggest that the online version of the ToSP is well suited to induce a state of high emotional activation or anxiety at least after the first announcement, that suggests that there is a chance to be exposed to distress screams. However, it appears that the induction of emotional activation during the second announcement was generally less effective in the Safety-Threat group. The emotional activation profile observed in the Safety-Threat condition also indicates a possible carry-over effect from the experience of the first safety announcement to the second threat announcement. Interestingly, the emotional activation pattern of the Threat-Safety group suggests that the emotional activation induced by a threat announcement might be reversible by a safety announcement.

### Recognition memory assessments

#### Recognition memory in the Safety-Safety and Threat-Threat groups

**Figure 3:**
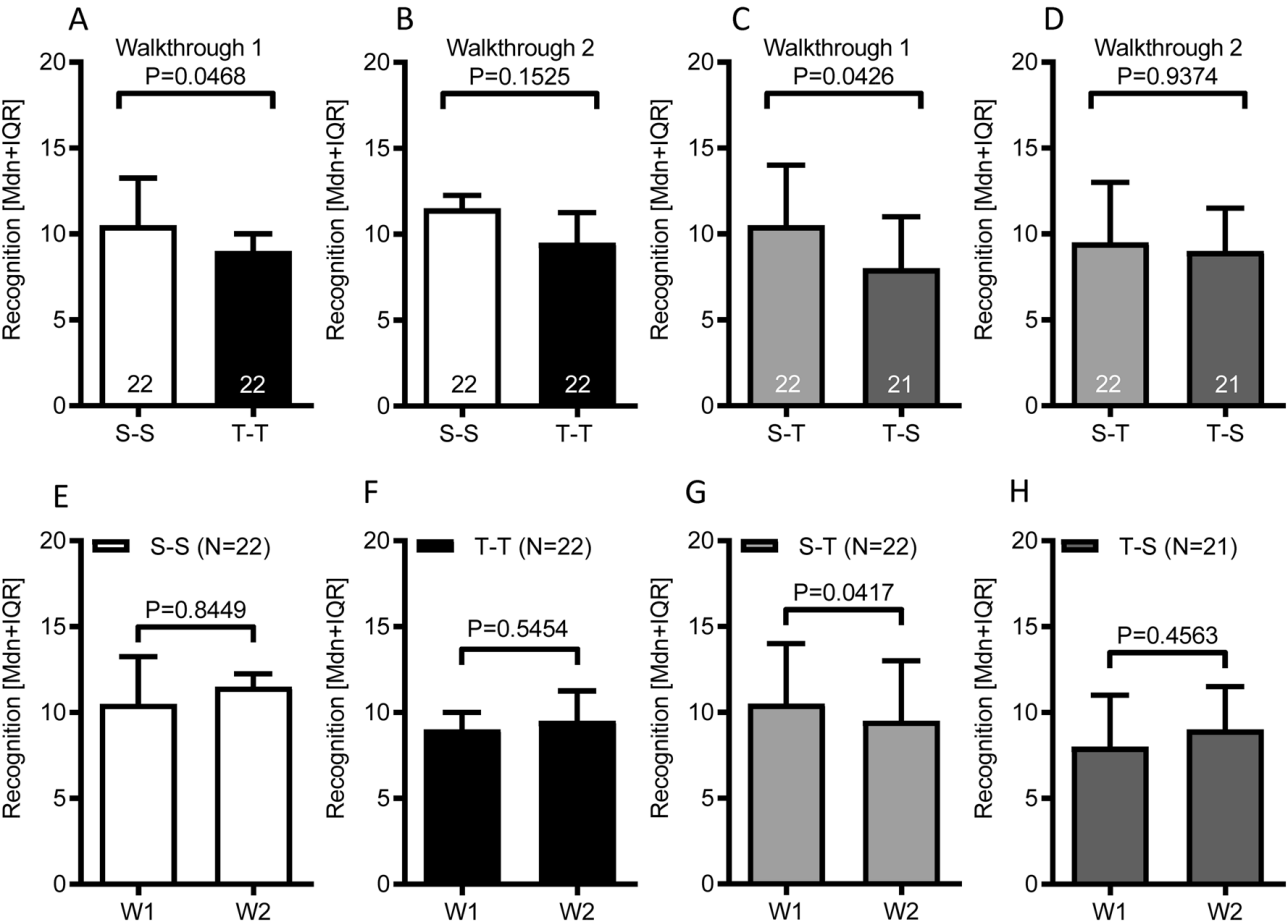
Recognition memory performance for information encoded either under threat or safety conditions. (**A, B**) Recognition memory performance for information encoded during the passive walkthrough 1 or 2 by the Safety-Safety and Threat-Threat experimental groups. Bars represents the median and interquartile range [Mdn + IQR] of total recognition scores. Numbers within the bars indicate the sample size of the experimental condition. *P*-values given refer to Mann–Whitney U test for independent samples. Abbreviations: S-S: Safety-Safety condition; T-T: Threat-Threat condition. (**C, D**) Recognition memory performance for information encoded during the passive walkthrough 1 or 2 by the Safety-Threat and Threat-Safety experimental groups. Bars represents the median and interquartile range [Mdn + IQR] of total recognition scores. Numbers within the bars indicate the sample size of the experimental condition. *P*-values given refer to Mann–Whitney U test for independent samples. Abbreviations: S-T: Safety-Threat condition; T-S: Threat-Safety condition. (**E–H**) Within-group dynamics of recognition memory performance for information encoded during the passive walkthrough 1 or 2. Bars represent the median and interquartile range [Mdn + IQR] of corresponding visual analog scale ratings. P-values given refer to Wilcoxon signed rank tests for dependent samples. Abbreviations: S-S: Safety-Safety condition; T-T: Threat-Threat condition; S-T: Safety-Threat condition; T-S: Threat-Safety condition; W1: Passive walkthrough video 1; W2: Passive walkthrough video 2.

The total recognition score for the first animated video was significantly lower in Threat-Threat group as compared to the Safety-Safety group (Passive walkthrough session 1: U = 158; *P* = 0.0468; Cohen’s *d* = 0.5071; effect size *r* = 0.2458; Mann–Whitney U test; Fig. 3A). No significant group difference was found for the total recognition score of the second animated video (Passive walkthrough session 2: U = 181; *P* = 0.1525; Mann–Whitney U test; Fig. 3B). Within-groups comparisons suggested that the recognition performance in both groups was similar for information retained from both animated videos (Passive walkthrough session 1 vs. 2: Safety-Safety: W = 10; *P* = 0.8449; Threat-Threat: W = 29; *P* = 0.5454; Wilcoxon test; Fig. 3E,F).

#### Recognition memory in the Safety-Threat and Threat-Safety groups

Similar to the finding for the Threat-Threat and Safety-Safety between-group comparison, the total recognition score for the first animated video was significantly lower in Threat-Safety group as compared to the Safety-Threat group (Passive walkthrough session 1: U = 148; *P* = 0.0426; Mann–Whitney U test; Cohen’s *d* = 0.6451; effect size *r* = 0.3070; Fig. 3C). However, no significant group difference was found for the total recognition score of the second animated video (Passive walkthrough session 2: U = 227.5; *P* = 0.9374; Mann–Whitney U test; Fig. 3D). It seems that the treat announcement prior to the second walkthrough session in the Safety-Threat group was less effective as compared to the threat announcement prior to the first walkthrough session in the Threat-Safety group. However, a within-group comparison performed for the Safety-Threat group on recognition performance for information retained from the first and second animated video, indicated significantly lower recognition scores for the second video (after the threat announcement), as compared to the first video (after the safety announcement), suggesting that the threat induction prior to the second walkthrough was not totally ineffective (Passive walkthrough session 1 vs. 2: Safety-Threat: W = − 93; *P* = 0.0417; Wilcoxon test; Fig. 3G). In contrast, the Threat-Safety group showed similar recognition performance for information retained from the first and second animated video (Passive walkthrough session 1 vs. 2: Safety-Threat: W = 25; *P* = 0.4563; Wilcoxon test; Fig. 3H).

The above results suggest that the encoding of complex event information, under the threat of distress scream exposure, reliably impairs recognition memory in the Threat-Threat and Threat-Safety conditions, specifically after a threat announcement prior to the first walkthrough session. The effect of the treat announcement prior to the second walkthrough session seems to be less prominent as evidenced by the recognition performance of the Safety-Threat group.

### Primacy vs. recency position effects on recognition memory

**Figure 4:**
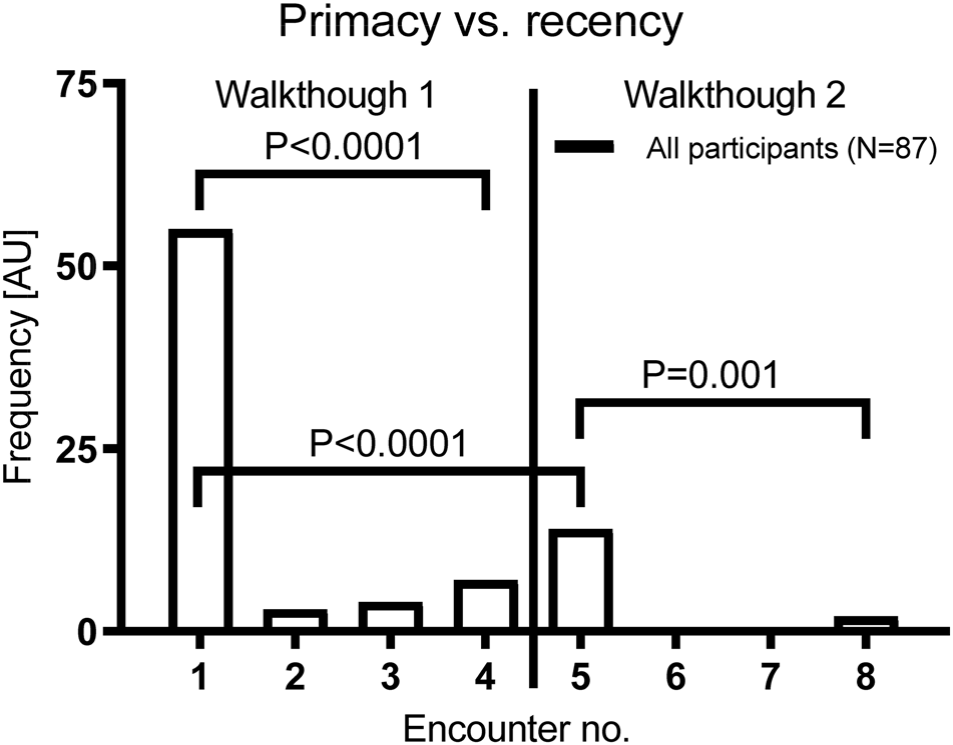
Primacy and recency position effects across all participants of the online study. Frequency distribution of a specific encounter (person target stimuli) spontaneously remembered. Encounter no. 1 refers to the first person encountered during the passive walkthrough 1 while encounter no 8 refers to the last person encountered during passive walkthrough 2. Bars represent the median and interquartile range [Mdn + IQR] of the frequency of the reported encounter. *P*-values given refer to Wilcoxon signed rank tests for dependent samples.

An across-groups analysis of the one encounter that was spontaneously remembered by the participants (without being cued with images from the walkthrough sessions), revealed that most participants recalled the first encounter from the first walkthrough session (63.2%), rather than the following encounters (Walkthrough session 1: Encounter 2 = 3.4%, 3 = 4.6%, 4 = 8%; Walkthrough session 2: Encounter 5 = 16.1%, 6 = 0%, 7 = 0%, 8 = 2.3; Fig. 4). A within-session analysis further revealed a significant primacy effect rather than a recency effect (Walkthrough session 1: Encounter 1 vs. 4: W = − 1176; *P* < 0.0001; Wilcoxon test; Walkthrough session 2: Encounter 5 vs. 8: W = − 66; *P* = 0.0010; Wilcoxon test; Fig. 4). The first encounter from walkthrough session 1 was recalled much more frequently as compared to the first encounter of the second walkthrough session (Encounter 1 vs. 5: W = − 861; *P* < 0.0001; Wilcoxon test; Fig. 4). Between-groups comparisons indicated that the frequency of the recall of the first encounter from walkthrough session 1 or 2 was not significantly different across the four groups (Encounter 1: χ2[3] = 3.830; *P* = 0.2804; Encounter 5: χ2[3] = 3.092; *P* = 0.3777 Kruskal–Wallis test; Data not shown).

These results suggest that the distractor task applied was indeed effective in blocking rehearsal and thus a recency effect with respect to the content of the passive walkthrough videos. The participants did not remember the last encounter in the second walkthrough more frequently as compared the other encounters from both the first and second walkthrough sessions. Therefore, it is unlikely that the recognition performance for the second walkthrough session was biased by differences in working or immediate memory. The recognition memory differences observed between the groups are therefore more likely to be mediated through differences in emotional activation during the encoding of the first passive walkthrough session rather than on group differences with respect to primacy or recency effects.

### Subjective time perception

The estimation of the length of the 2 walkthrough videos was not significantly different across groups (Time estimation walkthrough session 1: χ2[3] = 2.596; *P* = 0.4582; Time estimation walkthrough session 2: χ2[3] = 0.5733; *P* = 0.9025; Kruskal–Wallis test; Data not shown). These results suggest that the emotional activation induced by a threat announcement has no significant effect on the perceived length of the walkthrough videos that could be a manifestation of differences in encoding efficacy, which could account for the group differences in recognition memory reported above.

## Discussion

### Summary of findings

The results of our validation study suggest that the announcement of a risk to be exposed to distress screams apparently induces a level of emotional activation that is sufficient to impair the retrieval of complex information presented during passive walkthroughs in an animated scenario. Specifically, we found that threat of scream impaired complex multi-dimensional recognition performance that required the recognition of target person stimuli, the retrieval of spatial locations, temporal (which walkthrough session) and sequence order (room entries) information, as well as the emotional context (encoding under threat or safety conditions). Our results are the first demonstration of a threat of scream induced impairment of recognition memory in an animated context. To our knowledge, neither the ToSP nor the threat of shock paradigm have yet been systematically utilized to study complex learning and memory performance. In fact, up to date, there are only reports that threat of shock improves performance accuracy in a simple Go-NoGo task^34,35^ or modulates working memory performance^23,36–38^.

It remains to be determined, whether the online version of the ToSP would also be useful for the investigation of the relationship between experimentally induced anxiety and verbal and spatial working memory performance in healthy individuals as well as in patient populations with different anxiety disorders. The investigation of the relationship between pathological anxiety and working memory functions is especially interesting, since it has been shown that working memory training that is associated with a high memory load is capable to reducing anxiety symptoms in patients^39^.

Our results also suggest that the emotional activation induced by the threat of exposure to distress screams might be reversible after the announcement that there is no risk of being exposed to distress screams. This would potentially open the possibility to perform experiments with reversal designs where the participants can serve as their own controls^24^. However, it should be noted that the induction of emotional activation might be less effective if the threat announcement has been preceded by a no-risk safety announcement suggesting a possible carry-over effect specifically in the Safety-Threat condition.

It has been shown that a threat of shock can modulate working memory performance in an N-back task^37^. The implementation of a distractor task after the encoding trials successfully blocked the rehearsal (silent repeating or elaboration) of information, as suggested by the absence of a recency effect. Therefore, one can assume that the group differences in recognition memory were mediated by differences in emotional activation rather than as a consequence of immediate or working memory capacities.

By using a virtual reality-based approach, we have previously shown that patients with PTSD show impairments in spatial and temporal aspects related to episodic memory^8^. There is also evidence that experimental anxiety, induced by a threat of shock, leads to a pronounced underestimation of the duration of temporal intervals^40^. The perceived or estimated duration of a learning session might be correlated with the depth or quality of the encoding process, which in turn is predictive of the subsequent memory performance^32^. In this study, the emotional activation induced by the threat to be exposed to distress screams had no significant effect on the perceived length of the passive walkthrough videos. It remains to be determined, whether the subjective time estimation of the participants was indeed not influenced by the experimental threat vs. safety condition. From the present data, it remains unclear whether the estimation of the duration of the two videos was actually based on a feeling of how much time has probably elapsed or whether the estimations were mainly based on logical reasoning (e.g. the two videos must have had the same duration, since it was the same animated environment with four rooms and four persons in it).

Generally, our results suggest that the online version of the ToSP can be used to induce experimental anxiety outside of a psychophysiological laboratory. The paradigm might be used to investigate the effects of pathological anxiety, for example in patients with generalized anxiety disorder, on the encoding and retrieval of information. The ToSP could also be designed to assess possible changes in patients performance before and after therapeutic interventions in their home environment. This is especially suited for studies using internet interventions in the field of mental health. One major advantage of the online version of the ToSP is that it can reliably induce a state of anxiety without the necessity to pre-impose the punishing stimuli by force, and that it does not require the administration of the punishing stimuli during the encoding sessions to sustain the emotional activation. The application of the punishing stimuli during the encoding session would not only induce a sudden stress response but could instead perturb the encoding process. The online version of the ToSP has been devised to model pathological forms of anxiety as it is present in generalized anxiety disorder. These patients suffer from a constantly predominant feeling of threat that cannot be pinned down to a reasonable source. Frequently, the feeling of threat is rather diffuse and sometimes projected onto stimuli that are not perceived as threatening by healthy individuals^41^. Therefore, the administration of the punishing stimuli during the experiment would counteract the aim to model the effects of pathological anxiety on recognition memory.

The results of our study also argue against concerns that online studies generally require extremely large sample sizes to obtain significant effects of experimental manipulations. In the present study relatively small sample sizes of 21–22 individuals per group were sufficient to obtain statistically significant results.

The online version of the ToSP can also be used for research with participants or patients who are confined to a care facility, hospital, correctional facility or have a physical or mental disease (including agoraphobia) that impede a testing in the laboratory. It is also suited to design and conduct large-scale international studies with participants from different countries or continents. The availability of valid and reliable online paradigms to perform basic and clinical research is especially important during pandemic situations that impose high hygienic standards and protocols that are not compatible with some research subjects. Finally, the online version of the ToSP offers a unique opportunity to conduct longitudinal assessments of complex memory functions in populations at risk for the development of an anxiety disorder. Contrasting performance in the online version of the ToSP in healthy and (anxiety-) vulnerable populations might reveal boundaries between adaptive (e.g., response to threat) and maladaptive (e.g., pathological) effects of anxiety on distinct cognitive processes.

It should be also considered that online studies as compared to experiments performed in the laboratory, naturally, allow less control of the experimental situation. For example, the individual adjustment of the volume of the acoustic device has certainly generated some inter-individual variability. With respect to the distress scream stimuli used, their aversiveness is not fed by the volume intensity, but rather by their content transmitting a call for help, expression of pain, fear and despair and a social call into action. Normally, a social call into action cannot be easily ignored, because it is likely to be genetically preprogrammed response. Therefore, the loudness might not have been a decisive factor in the effectiveness of the distress stimuli.

The threat of scream paradigm is used to experimentally induce a state of anxiety that is based on an unpredictable threat. After the announcement “You are now at risk to be exposed to distress screams”, participants cannot predict with certainty whether they will be exposed to a distress scream or not. It has been shown that an unpredictable threat induces an elevated startle eye-blink response, as compared to a predictable threat^42^, suggesting that the threat-induced emotional activation that modulates the responsiveness of defensive reflex systems is much more pronounced during unpredictable vs. predictable threat. It has been proposed that unpredictable threat induces a state of anxiety that is associated with behavioral and cognitive avoidance, facilitation of defensive responses and hypervigilance, while predictable threat in contrast induces a state of fear that goes along with behavioral responses of fight, flight, or behavioral freezing^43^. These different emotional states of fear and anxiety might also involve different neural substrates^44^. A heightened sensitivity to unpredictable threats might increase the susceptibility to develop anxiety disorders^42,45–48^. It remains to be determined whether the threat of scream paradigm could be utilized as a screening device for individuals at risk to develop an anxiety disorder.

## Conclusions

The online version of the ToSP is a valid and reliable tool to model the disabling effect of pathological anxiety on learning and memory performance. To this end, the paradigm could also be used to investigate the effects of threat of scream-induced emotional activation on basic cognitive and pathological processes including perception, attention, concentration, decision making, avoidance behavior, impulsivity and compulsive behavior. It could also be used to evaluate the efficacy of therapeutic interventions on the ability to cope with feelings of stress and anxiety to reinstate normal cognitive function.

## Supplementary Information

Supplementary Information.

## Data Availability

The datasets generated during and/or analysed during the current study are available from the corresponding author on reasonable request.
