# Supplementary material for: Recognition memory, primacy vs. recency effects, and time perception in the online version of the fear of scream paradigm
Source: Sci Rep. 2022 Aug 22;12:14258. doi: 10.1038/s41598-022-18124-9 (PMC9395394; doi:10.1038/s41598-022-18124-9)
Supplement: Supplementary file 1 — Supplementary Information. [file 41598_2022_18124_MOESM1_ESM.docx]

**Online Supplement**

**Distribution of the time required by the participants to complete the task**

Figure S1 shows the frequency distribution of the time required by the participants to complete the task. The frequency distribution appears as spread with a modest positive right skew. Participants that had completed the task in less than 25 minutes or took longer than 90 minutes are considered as outliers and have been excluded form data analysis.


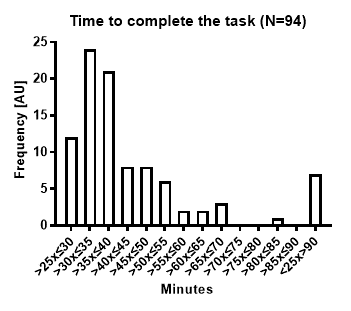


Figure S1. Frequency distribution of the time required to complete the task. Each bar represents the number of participants in the indicated time intervals.

**Normality check and justification of non-parametric data analysis**

We used non-parametric tests because the data was generally not normal distributed. The figure S2 shows QQ normality plots for the 4 conditions in which the values predicted by a Gaussian distribution are compared to the actual values for the manipulation check data. The P-values indicated refer to Kolmogorov-Smirnov tests performed with baseline, announcement 1 and announcement 2 data. Only 3 out of 12 data sets followed undoubtedly a normal distribution. Therefore, it seemed not justified to use parametric statistics.


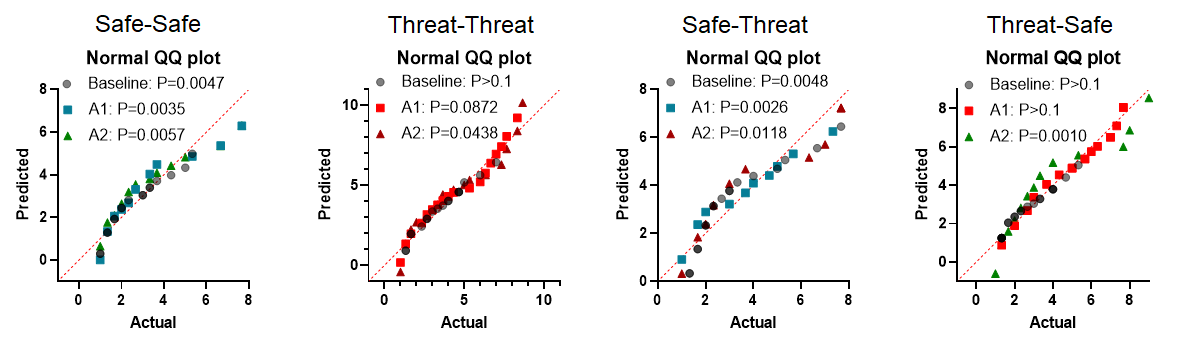


Figure S2. Results of normality tests. QQ plots illustrate the discrepancy between expected values and the raw data obtained.

**Sample size justification and power determination**

In the course of a follow-up experiment we recruited a new cohort of 22 participants, out of which one participant was excluded as an outlier (baseline: # of values: 22, z:3.24, critical value of z:2.7577; p<0.05; Grubbs-test). The figure S3 shows that threat-induction induced a significantly higher subjective anxiety, stress and arousal response as compared to the baseline condition (N=21; Baseline: mean: 5.333, SD: 2.221; Threat: mean: 8.571, SD: 4.843; T(df:20):3.558;P=0.0020; T-test for dependent samples). We used this manipulation check data set to calculate the minimal sample size required to obtain an effect of similar size using G*Power. The power analysis yielded a minimal sample size of N=20 (Baseline and Threat: N=21; Input parameters: one-tailed, effect size dz: 0.7711450, α error probability: 0.05, power: (1-β error probability: 0.95; Output parameters: Non-centrality parameter δ: 3.4486653, critical t: 1.7291328,df: 19, total sample size: 20, actual power: 0.9532514). Based on this power analyses we are confident that the sample sizes in the present study have sufficient power to protect against false negative results.


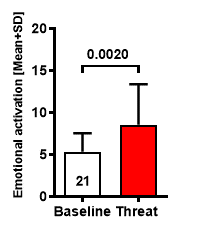


Figure S3. Emotional arousal before (baseline) and after threat induction. Bars represents the mean and standard deviation of subjective anxiety, stress and arousal ratings made on visual analog scales. P-value given refers to a t-test for dependent samples.

The figure S3 shows successful induction of emotional arousal after threat-induction in a new cohort of healthy participants (N=21). Bars represent sum scores of subjective ratings (on visual analogue scales) of the current level of anxiety, stress and emotional arousal prior to the start of the experiment (Baseline), during the baseline measurement and after the threat-induction.

**Replication experiment**

We performed a follow up online experiment with an N = 85 participants and the same four conditions. We obtained similar results with respect to subjective emotional arousal ratings (see figure S4 below). We are therefore confident that the sample sizes used in the present study are sufficiently large to investigate the effects of threat-induction on cognitive performance and that the risk to generate false negative results due to insufficient power is rather low.


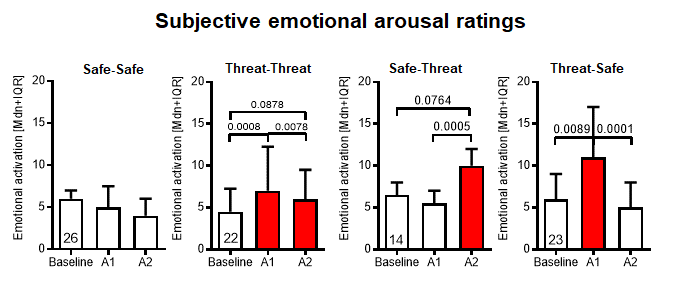


Figure S4. Emotional arousal before (baseline) and after threat announcements for the indicated conditions. Bars represents the median and interquartile range of subjective anxiety, stress and arousal ratings made on visual analog scales. P-value given refers to Wilcoxon signed rank tests for dependent samples. Abbreviations: S-S: Safety-Safety condition; T-T: Threat-Threat condition; S-T: Safety-Threat condition; T-S: Threat-Safety condition.

The figure S4 above shows the results of a follow-up study in which experimental anxiety was successfully induced in a separate cohort of healthy participants (N=85). Figures show sum scores of subjective ratings (on visual analogue scales) of the current level of anxiety, stress and emotional arousal prior to the start of the experiment (Baseline), after the first or second announcement of threat or safety conditions. Each data set shows within-group dynamics of emotional activation during the baseline measurement and after threat or safety announcements for the four experimental conditions.
